# Precision diagnostic and therapeutic interventions in rare genetic neurodevelopmental disorders

**DOI:** 10.1038/s41390-025-04611-y

**Published:** 2025-11-22

**Authors:** Anahid A. Assadourian, Julian A. Martinez-Agosto

**Affiliations:** University of California Los Angeles, Los Angeles, CA USA

## Abstract

**Abstract:**

Neurodevelopmental disorders (NDDs) include a broad spectrum of phenotypes spanning from intellectual disability (ID) to developmental delay (DD) and autism spectrum disorder (ASD). As neurodevelopmental phenotypes are a common presenting feature of an underlying genetic condition, professional medical organizations recommend genetic testing for all individuals with a NDD. When testing is pursued, identified genetic differences can lead to personalized clinical management with early diagnosis supporting the development of surveillance and intervention for co-occurring adverse health outcomes. Despite this, barriers to testing have prevented individuals from receiving a genetics referral and testing. Current therapeutic modalities including small molecule drugs, gene therapies, and antisense oligonucleotide therapies have emerged and shown promise in preclinical trials with therapeutic drugs gaining FDA approval. However, translational challenges are extensive, especially for identifying biomarkers of drug effects in the CNS. In this review, we discuss diagnostic approaches and clinical utility of genetic testing for rare genetic neurodevelopmental disorders, emerging development of individualized therapies, and progress for current therapeutics in addition to challenges with clinical translation and delivery. We will highlight opportunities for early diagnosis and treatment that are steadily gaining ground in favor of optimizing long-term health outcomes and improving quality of life for neurodiverse individuals.

**Impact:**

- The path from genomics to therapeutics for neurodevelopmental disorders continues to present multiple opportunities and challenges.
- While emerging genome-wide sequencing and gene editing technologies deliver increased diagnostic yields and alternatives to life-long small molecule therapies, clinical translation has been challenging due to inherent cost and genetic heterogeneity.
- Limited access to genetic testing despite practice guidelines remains a barrier towards precision therapeutics for rare neurodevelopmental disorders, while pre-clinical investigations face obstacles when translating to human subjects.
- This review will summarize the impact of existing successes in diagnosis and therapeutics for neurodevelopmental disorders while highlighting ongoing challenges and areas of future opportunities.

## Introduction

Neurodevelopmental disorders (NDDs) represent a diverse spectrum of phenotypes ranging from intellectual disability (ID) to behavioral differences. Defined as delays in expected timelines for developmental milestones, developmental delay (DD) presents early in childhood, and when persistent and coupled with cognitive impairment by the age of 5 yo, often precedes an ID diagnosis. In a subset of individuals with DD, behavioral differences that affect social interactions establish a diagnosis of autism spectrum disorder (ASD). Ranging in presentation and severity with onset as early as the first year of life, core autism characteristics include enduring challenges in social and communication development alongside restrictive, repetitive behaviors.^1^ Common comorbidities between ASD and behavioral and psychiatric disorders such as anxiety and attention deficit hyperactivity disorder (ADHD) have been well-evidenced in the growing literature.^2^ For subsets of autistic individuals more significantly affected, additional symptoms may extend beyond behavioral differences to include cognitive and motor deficits, DD, as well as medical comorbidities.

With rising prevalence in the past two decades, ASD approximately affects 1 in 36 children in the United States according to the Centers for Disease Control and Prevention’s Autism and Developmental Disabilities Monitoring (ADDM) Network.^3^ Heritability estimates support ASD as the most heritable NDD,^4^ based on large differences in concordance rates between monozygotic and dizygotic twins, with monozygotic twins having rates approximately three times higher than those of dizygotic twins.^1^ These population-based twin studies have consistently observed a strong genetic correlation between autism traits and other neurodevelopmental diagnoses.^5^ Increased prevalence of clinically significant autism characteristics have similarly been shown in individuals with genetic syndromes associated with ID compared to individuals in the general population.^6^

Intellectual disability is characterized by early-onset below-average intellectual functioning in addition to noticeable limitations to adaptive functioning.^7^ ID typically presents alongside global DD, defined as a delay in two or more developmental domains such as motor, speech, and cognition; many children with ID are initially diagnosed with global DD.^8^ Individuals with ID have a higher prevalence of physical conditions such as neurological disorders, congenital malformations, and sensory impairments when compared to the general population.^9^

NDDs manifest with vast phenotypic and genetic heterogeneity that extends across presentations and genetic syndromes. An emerging distinction in their phenotypic presentation is the likelihood of an underlying genetic diagnosis depending on the presence of distinct signs or phenotype combinations that help to identify underlying genetic conditions. The likelihood of an associated genetic diagnosis increases with additional co-occurring medical diagnoses, in contrast to cases with isolated neurodevelopmental phenotypes.^10^ It is estimated that up to 40% of autistic individuals with complex phenotypic presentations may carry a diagnosis of a genetic syndrome or chromosomal abnormality.^11^ Similarly, a genetic diagnosis is more likely in cases of ID that present associated with congenital malformations or comorbidities, or additional clinical features such as epilepsy and metabolic manifestations, compared to those that manifest ID or global DD as the only presenting feature.^8^

One of the major findings and subsequent challenges in identifying risk genes for NDDs is that most of the risk, at the population level, derives from common genetic variation.^12^ Genetic changes that are causally linked to disease and are thus rare in the population remain missing in larger-scale genomics data. However, rare genetic changes have recently emerged repeatedly across patients through informed investigations of shared similar symptom profiles. As ASD and ID are often a presenting feature of a genetic condition, professional medical organizations such as The American College of Medical Genetics and Genomics (ACMG) initially recommend that all individuals with an ASD diagnosis be offered genetic testing, with chromosomal microarray (CMA) testing suggested as first-tier testing for all patients and Fragile X testing suggested for males.^11^ In cases where genetic testing is pursued, an identified genetic diagnosis can lead to clinical utility, with early diagnosis supporting the development of personalized surveillance and intervention plans. Development of a personalized treatment plan based on precise genetic findings can likewise aid clinicians in anticipating the development of associated co-occurring conditions.^13–20^ However, for many genetic disorders that fall beyond the classification of rare to ultra-rare, limitations remain on informed clinical management because of their scarcity in the population which creates a paucity of natural history data.

**Fig. 1:**
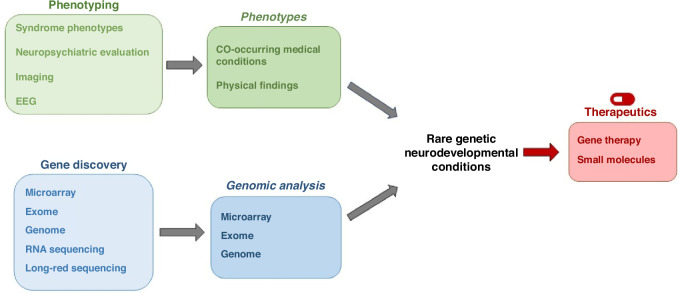
Integration of phenotyping and genotyping technologies into clinical diagnostics and evaluations translates to therapeutic advances for rare genetic neurodevelopmental conditions.

In this review, we will discuss the diagnostic approaches and clinical utility of genetic testing in early diagnosis of rare genetic NDDs and emerging development of individualized precision therapies. This review will integrate existing successes in diagnosis and therapeutics for rare genetic NDDs while highlighting ongoing challenges and areas of future opportunities (Fig. 1). We emphasize the application of these recommendations in identifying and treating the medical comorbidities that disproportionately affect individuals with NDDs in favor of optimizing long-term health outcomes and improving quality of life.

## Gene discoveries and translational advances define rare neurodevelopmental disorders

Over the past decade a flurry of discoveries has expanded the number of novel single gene genetic conditions associated with neurodevelopmental phenotypes.^13^ Advances in genome sequencing technologies, coupled with improvements in reference genomes and databases of natural population variants, have led to the identification of thousands of rare and ultra-rare genetic disorders.^21,22^ Emergence of genome-wide sequencing tools and their improvements over time has iteratively increased the spectrum of genomic alterations associated with human phenotypes.^23^

Early on, genetic testing was restricted to evaluations of chromosome number and structures (ploidy) using karyotypes, coupled with fluorescence in situ hybridization for detection of deletions and duplications of chromosomal segments.^24^ This led to the recognition of fragile sites on the X chromosome and the identification of the FMR1 gene associated with Fragile X syndrome, one of the most common NDDs in males.^25^ Chromosomal microarray allowed for the detection of smaller losses and gains of chromosomal material (copy number variations, CNVs) and revealed many common genetic etiologies now commonly associated with ASD and ID, such as deletions or duplications in 1q21.1, 7q11.23, 15q11.2, 16p11.2 and 22q11.2.^26–28^ Characterizing single gene contributions to NDDs, however, proved more challenging, as it often required large families (pedigrees) of affected individuals to link a specific gene to the observed NDD (linkage analysis). The advent of next generation sequencing (NGS), the ability to massively sequence all regions of the genome in parallel, allowed for the generation of complete genomes and thus identification of all protein-coding genes.^29^ This disruptive technology allowed for expansion of the known repertoire of single nucleotide gene variants (single nucleotide variations) known in the population.^30,31^ NGS can be used to sequence a whole human genome (genome sequencing, GS), or by capturing only the gene sequences that code for proteins (exome), generate whole exome sequences (exome sequencing, ES) to identify variants that are distinct from reference genomes.^32^ As more genomes were sequenced in unaffected individuals, collection of the data in centralized databases like the Genome Aggregation Database (gnomAD^33^) allowed for population frequency estimates for each single nucleotide variant (SNV), establishing prevalence in the general population. These efforts have facilitated identifying SNVs rarely observed in the population, which statistically represent gene variants under negative selection (not passed down from generation to generation).^22^ It is these rare variants that, in combination with the shared phenotypes observed across individuals that carry them, have established new diseases and genetic syndromes associated with NDDs.^34^ Analysis of family trios, in which ES for both unaffected biological parents is included in the variant analysis, has allowed identification of de novo variants only present in the affected proband as one of the most common diagnostic etiology.^35^ All of the above-mentioned genomic sequencing technologies have successfully transitioned as diagnostic tests with demonstrated impact on identifying genetic associations for greater than 40% of syndromic NDDs.^15^

### Genetic heterogeneity

Genome-wide diagnostic tests implemented in the clinical care of individuals with NDDs include chromosomal microarray, ES, and GS. Genome-wide chromosomal microarray screens the entire genome for CNVs and has been recommended as a first-tier test among patients with NDDs.^13^ CMA technologies can detect CNVs, but face limitations in the size of the deletion or duplication that can be detected. In the last decade, technologies that have enabled sequencing large amounts of DNA have emerged, allowing for testing beyond single or several genes to test panels that include extensive gene lists. In ES, exons of the genome are sequenced facilitating the detection of sequence level, single gene variants, while GS allows for comprehensive assessment of both CNVs and single gene variants across the genome.

The phenotypic heterogeneity of NDDs extends to their genetic architecture as recent analyses utilizing modern genome scanning have identified hundreds of genes worthy of further investigation. ES studies have revealed an estimated 400–1000 genes to be involved in ASD susceptibility,^36^ with studies of large genotyped cohorts having recognized several pathogenic chromosomal copy number variants (CNVs) as major contributors of risk.^37^ Prevalence of pathogenic CNVs are estimated to be 10 to 15% in individuals with ASD.^38^ Although rare versus common genetic variants continue to be studied in their contributions towards autism risk, common variation contributes significantly at the population level.^39^

### Phenotypic heterogeneity

Approximately 80% of ASD cases are non-syndromic with no clear connection to a single genetic condition.^40^ However, growing research studies on the genetic architecture of ASD have supported that different individuals with the same or similar clinical presentations may have varying and distinct underlying genetic etiologies.^41^ Thus, co-occurring symptoms in combination with an ASD or DD diagnosis suggest that a genetic condition should be considered within these populations.

An ASD or NDD diagnosis accompanied by additional medical comorbidities is highly suggestive of underlying genetic conditions. Studies have measured the incidence of cancer in ASD to be globally equal to that of the general population,^42^ highlighting cancer as an additional phenotype not directly associated with autism and its related mechanisms. However, considered rare in combination, an ASD diagnosis with such medical findings should prompt a patient’s referral to a geneticist. Lysosomal storage disorders (LSDs) are metabolic disorders characterized by reduced or absent levels of lysosomal enzymes, leading to accumulation of macromolecules and resulting in organomegaly. In individuals with an ASD diagnosis, medical concerns aligning with distinct presentations seen in patients with LSD should, likewise, lead to a genetic evaluation. The presence of neurodegenerative symptoms, characterized by developmental regression or progressive loss of milestones, alongside ASD are further examples of potential co-occurring diagnostic concerns. In the setting of individuals with diagnosed DD or ASD, presenting with symptoms of regression unique to known genetic syndromes should prompt physicians to seek genetic evaluation and testing.

Early-onset, concomitant neurodevelopmental and medical diagnoses can serve as key markers for identifying an associated genetic condition and, in some instances, are amenable to screening and intervention. Significant motor differences can be exhibited in infants with elevated likelihoods of autism between 6 and 36 months of age, including delays in reaching motor milestones as well as fine and gross motor problems.^43^ In a retrospective review of 316 patient charts in a neurodevelopmental clinic, hypotonia and motor delays emerged as being strongly associated with a genetic diagnosis, with an approximately 5 to 11% increase in likelihood for an associated pathogenic variant with each 1 month delay in walking onset.^44^ Motor phenotypes have thus emerged as highly predictive of an associated genetic condition, highlighting cases where genetics diagnostics may be pursued even when a corresponding neurodevelopmental diagnosis is missing. Similarly, a common association has been recognized in children with epileptic encephalopathies (such as infantile spasms) and ASD for tuberous sclerosis complex (TSC),^45^ a genetic disorder characterized by the development of benign tumors across several organs.^46^ Congenital anomalies and cardiomyopathies are among additional co-occurring phenotypes that, in a patient diagnosed with a NDD, should trigger a referral for a genetic evaluation as emerging therapeutic opportunities depending on genetic diagnosis, particularly genes associated with the RAS signaling pathway (rasopathies), are having an impact on health outcomes.^47–49^

Broad heterogeneity in phenotypic presentation across genes creates additional complexities when considering syndrome specificity and developing informed interventions. Individuals with a diagnosed genetic condition may not present with the same symptoms despite having identical genetic etiologies. This can be due to differences in penetrance, the likelihood of observing a specific phenotype associated with a genetic condition, or variable expressivity, the variety of symptoms observed in individuals affected by a specific genetic condition. An example is TSC, which presents both genetic heterogeneity and variable expressivity. Tuberous sclerosis complex can be caused by either the TSC1 or TSC2 genes, and individuals carrying the same disease-causing gene variant within or between families might present with a variable spectrum of phenotypic findings.^50^ Considering this diversity and reduced penetrance within groups of dominant genetic diseases, recommendations for genetic testing may require to be bolder and more expansive, encompassing all individuals with a known NDD as this diagnosis, even in isolation, may represent a milder and earliest manifestation of an otherwise more complex genetic syndrome.

### Diagnostic outcomes and gene networks

To date, there are more than 3000 genes with unique associations to neurodevelopmental phenotypes as part of a genetic syndrome. As more gene variants are discovered associated within each of these genes, current practice guidelines direct their classification into three major categories: pathogenic/likely pathogenic, benign, and variants of uncertain significance.^51^ A positive diagnostic test result is usually associated with a pathogenic or likely pathogenic variant in a well-established gene associated with neurodevelopmental symptoms. A negative result does not report any variants for the patient; however, this does not rule out the possibility of a genetic association. Typically, additional research needs to take place for the number of genes associated with NDDs to expand. Therefore, patients are usually recommended to follow up over the years as more genes are identified and more comprehensive tests are developed. Finally, variants of uncertain significance present a unique challenge to patients as they typically are neither associated with pathogenic or benign status. This also requires follow-up over time and additional research to take place to eventually resolve the variant uncertainty. Furthermore, current gene variant reference databases lack comprehensive diversity, which increases the likelihood of reporting variants of uncertain significance in instances where those variants are prevalent in specific ancestries that are not represented in the databases.^52^

For those with a pathogenic variant, the genes associated with NDDs have emerged as important in several developmental processes during brain formation as well as development and function of other organs. Large ASD genetic screening studies have identified broad categories of gene pathways that have been identified recurrently across NDDs. Amongst these, chromatin modifying proteins and signaling pathways have emerged as the most common and most likely targetable genetic associations. Dysfunction in the ERK/MAPK signaling pathway has been implicated in numerous NDDs including ASD, ADHD, and Fragile X syndrome, with mutations in the signaling machinery correlating to both upregulation and downregulation of proteins that make up the ERK/MAPK cascade.^53^ Additionally, the PI3K/mTOR pathway has emerged with a central role in numerous developmental processes associated with autism as mutations of several genes involved in components of the pathway such as *TSC1* and *PTEN* are required for crucial roles in protein synthesis and mRNA translation, all common cellular phenotypes that when altered are associated with ASD.^54^

As a result of many of these gene families and their co-occurring conditions emerging as frequent associations with NDD, many patient-focused organizations as well as academic institutions have developed clinics that provide care to patients with these rare conditions. Examples include Centers for Excellence for Angelman syndrome and Tuberous Sclerosis supported by private foundations like the Tuberous Sclerosis Alliance. Similar clinics that focus on chromatinopathies, metabolic disorders, or are genetic syndrome-specific have emerged across medical centers to provide precision care unique to these conditions. Providing access to highly trained specialists for specific conditions and a genetics-first approach to care, the UCLA Care and Research in Neurogenetics (CARING) Clinic serves as one such model for delivering neuropsychiatric care for patients.^55^ Among 316 patients over a 5-year period, approximately 78% received a genetic testing before, during, or after CARING clinic and, of the 152 patients with an identified pathogenic or likely pathogenic variant, 36 patients received their genetic diagnosis from the CARING Clinic. The clinical management of all 36 patients with pathogenic or likely pathogenic variants was also affected in at least one domain. Clinics that utilize a genomics-informed approach to care agnostic to a specific diagnosis provide significant clinical benefits to patients with NDDs, directly affecting management across multiple domains. As the number of genetic syndromes associated with NDDs expands over time, emphasizing care around core principles of genomic medicine over individual syndromes will likely become the norm.

### Barriers to access to genetic testing

Although genome-wide technologies have translated into the clinic as genetic diagnostic tools, there remain multilevel barriers to access that prevent affected individuals from receiving genetic testing. An empirical study examining parental experiences and challenges with pathways to ASD genetic testing highlighted a broad lack of insurance coverage as discouraging many providers from ordering testing, simultaneously raising concerns about the utility of such testing.^56^ Of the remaining 20% of patients who did not receive genetic testing, these individuals were similarly reported to have been denied by insurance for not meeting specific insurance policy requirements for testing, or to have been lost to follow-up.^55^ Insurance denial rates can be as high as 18% in many instances.^57^ Additionally, many patients and their families may never receive access to a specialist who can recognize certain medical concerns as requiring additional testing, either because of their specific policy coverage or through geographic barriers preventing access to large academic and clinic centers. These geographic barriers include lack of transportation, concentration of specialists in urban centers, lower health literacy, digital divide, and lack of primary care provider knowledge.^58^ Lack of knowledge amongst providers on what tests to order and when, as well as how to interpret genetic test results, likewise remains a significant barrier to patients gaining a genetic testing referral.^59^ Perhaps most challenging are perceptions that genetic testing is unlikely to affect clinical management. However, evidence-based guidelines and position statements have demonstrated the clinical utility of a referral to a genetics provider and genetic testing.^60^

## Clinical utility of genetic testing

Identification of an individual’s underlying genetic diagnosis can provide both personal and clinical utility to patients and their families.^61^ The utility that arises from genetic testing, however, varies in many ways from other diagnostic techniques. As patients undergo medical testing and receive a diagnosis, the subsequent expectation is to receive an intervention for their condition. The complexities of a genetic diagnosis are exemplified here, in that there may not be an identified, direct action to intervene. Thus, the question of clinical utility is better answered when considering the potential benefits patients experience from receiving an early diagnosis of a genetic condition. Beyond providing a diagnostic answer (which many patients and their families find valuable in and of itself as the end of a diagnostic journey), a genetic diagnosis can lead to positive changes in surveillance, medical management, and access to clinical trials/research for individuals with rare genetic conditions.^38^ For example, a genetic diagnosis of GLUT1 deficiency can lead to the implementation of a therapeutic intervention like the ketogenic diet. In a systematic evidence-based review of literature describing the clinical outcomes resulting from ES and GS in patients with congenital anomalies, DD, or ID, 95% of the 167 included studies reported a change in the clinical management of patients.^61^ Genetic counseling as part of the diagnosis process can also have an impact on patient decision-making and family planning.^62,63^ Additionally, access to support and advocacy groups as well as the potential for reduced healthcare costs highlight some of the personal impacts patients and their families may experience. As research continues to be generated regarding the best practices and guidelines for the medical management of rare genetic NDDs, in addition to the development of precision therapies, rates of changes in clinical management can be expected to increase as a result of patients receiving an early genetic diagnosis.

## Therapeutic approaches to rare genetic neurodevelopmental disorders

Advances in therapeutic approaches for rare genetic NDDs have seen varying levels of success in distinct patient populations. Small molecule drugs have been a primary focus for developing therapeutics due to their ease of delivery and demonstrated high efficacy in specific conditions.^64^ Through oral delivery in the form of a pill, such small molecules can penetrate cell membranes and modulate the function of specific enzymes and receptors, allowing for potentially therapeutic effects by directly or indirectly targeting the altered protein or signaling pathway. However, most current treatments target symptoms rather than underlying pathophysiology. Alternatively, gene therapies have emerged as a novel technique with the potential to reverse genetic abnormalities underscoring disease pathogenesis.

Natural history studies have emerged as an essential component in the development and approval of therapeutic interventions for rare diseases. These studies collect longitudinal phenotypic data to identify specific clinical endpoints amenable to inclusion in clinical trials to facilitate drug development and approval.^65^ For gene therapy to be a viable treatment option, the condition must have a well-established natural history with the potential for correction through the administration of functional genes copies or reversion of sequence variants; thus, NDDs generally associated with highly penetrant single gene variants, such as Angelman syndrome, are the ideal candidates for gene therapeutics.^66^

Delivery methods and administration routes are a few of the current barriers when considering the therapeutic approach most amenable for treatment in rare genetic conditions. The use of small molecule drugs is characterized by short-term effects, requiring continuous oral intake of the drug across an individual’s lifetime. In contrast, gene therapies provide long-term effects through a single dose but require delivery via an injection into the body. Despite systemic injection being a suitable and attractive option for delivery in cases where the genetic condition is characterized by genetic abnormalities outside of the brain, low blood-brain barrier (BBB) permeability creates additional obstacles with injection delivery for central nervous system (CNS) disorders.^67^ For such cases, an intrathecal injection would be administered to bypass the BBB, allowing the therapy to reach the CNS directly. The complexities involved in the delivery of therapeutics to the brain similarly impacts the efficacy of small molecule drugs, as success rates of CNS drug development remains at 8% in comparison to rates for cardiovascular drugs at approximately 20%.^68^ Ensuring the ability of a drug to cross the BBB is one of the major barriers limiting the use and efficacy of these medications. A number of emerging approaches are currently under development to overcome this barrier, including nanoparticle carriers, receptor-mediated transcytosis, focused ultrasound, cell-penetrating peptides, and intranasal delivery.^69–71^

### Outcome measures and biomarkers guiding intervention

The development and implementation of therapeutic interventions relies largely on the identification of outcomes that can be measured accurately and reproducibly amongst individual patients, providing quantitative and objective metrics of relevant clinical processes.^72^ While treatments are continually developed for many genetic NDDs, relevant clinical outcome assessments and biomarkers have been limited. In more recent years, electroencephalography (EEG) has been used as a potential diagnostic or translatable biomarker in clinical translational studies of genetic NDDs as well as idiopathic ASD, with qualitative analyses of EEGs in patients with Angelman syndrome revealing rhythmic delta activity as a reliable EEG biomarker.^73^ Additionally, protein and peptide-based biomarkers in ASD have been an emerging focus with studies highlighting the analysis of both protein expression levels and posttranslational modifications as having potential to increase the specificity and accuracy of markers.^74^ Similarly, MRI-based assessments are emerging as a reliable biomarker for clinical trial response.^72^

Behavioral assessments have similarly been used as primary methods for assessing therapeutic trial efficacy endpoints when administered pre and post intervention. The Autism Diagnostic Observation Schedule (ADOS) is a formal assessment with a standardized, semi-structured approach to evaluate social and communication skills, play, and creativity for individuals suspected of having ASD.^75^ When evaluating the efficacy of targeted behavioral interventions for individuals with an ASD diagnosis, re-administering the ADOS may serve as an evaluation of behavioral changes that are clinically significant. Alternatives to the ADOS include the Vineland Adaptive Behavior Scale and the Repetitive Behavior Scale-Revised and Social Responsiveness Scale.^76^ However, the vast majority of outcome measures lack reliability, validity, and sensitivity to treatment, as evidenced by multiple failures in Fragile X syndrome therapeutic trials.^77^ As such, the identification and use of objective outcome measures and biomarkers is of paramount importance when developing therapeutic interventions to establish mechanisms of evaluating change.

### Existing and emerging therapeutic interventions for genetic neurodevelopmental disorders

As previously discussed, the extensive phenotypic heterogeneity in genetic NDDs presents challenges for identifying reliable endpoints for efficacy in the development of therapeutic interventions. However, advances in small molecule drugs and gene therapies offer promising approaches to treating co-occurring conditions in these populations. While not disease modifying, small molecule therapies have shown great potential in alleviating specific symptom domains, such as seizures, and having a large impact on quality of life.^78^ In the past decade, a few small molecules and gene therapies have been developed and FDA-approved for the treatment of genetic disorders (Table 1). Despite only a few, several examples of these successes have been recently reported for specific genetic syndromes associated with NDDs.

**Table 1:** Therapeutics for genetics conditions approved from 2014-2024 by the Food and Drug Administration (FDA)

Drug (brand name)	Mechanism of Action	Drug Indications	Year of FDA Approval
Elosulfase alfa (Vimizim)	Hydrolytic lysosomal glycosaminoglycan-specific enzyme	Mucopolysaccharidosis type IVA	2014
Metreleptin (Myalept)	Leptin analogue	Leptin deficiency in patients with congenital or acquired generalized lipodystrophy	2014
Suvorexant (Belsomra)	Orexin-receptor antagonist	Insomnia	2014
Eliglustat (Cerdelga)	Glucosylceramide-synthase inhibitor	Gaucher’s disease	2014
Cholic acid (Cholbam)	A primary bile acid	Bile acid synthesis disorders and peroxisomal disorders	2015
Ivacaftor plus lumacaftor (Orkambi)	CFTR potentiator plus CFTR corrector	Cystic fibrosis in patients with homozygous DF508 *CFTR* mutation	2015
Sonidegib (Odomzo)	Smoothened inhibitor	Basal cell carcinoma	2015
Uridine (Xuriden)	Pyrimidine analogue	Hereditary orotic aciduria	2015
Asfotase alfa (Strensiq)	Alkaline phosphatase	Hypophosphatasia	2015
Cobimetinib (Cotellic)	MEK inhibitor	Melanoma with BRAF_V600E/K_ mutations	2015
Sebelipase alfa (Kanuma)	Enzyme replacement therapy	LAL deficiency	2015
Eteplirsen (Exondys 51)	ASO designed to target dystrophin pre-mRNA	Duchenne muscular dystrophy	2016
Nusinersen (Spinraza)	ASO designed to target SMN2 pre-mRNA	Spinal muscular atrophy	2016
Deflazacort (Emflaza)	Corticosteroid	Duchenne muscular dystrophy	2017
Deutetrabenazine (Austedo)	VMAT2 inhibitor	Chorea associated with Huntington disease	2017
Vestronidase alfa (Mepsevii)a	Recombinant human lysosomal β-glucuronidase	Mucopolysaccharidosis VII	2017
Emicizumab (Hemlibra)a	Bispecific FIX and FX-directed antibody	Hemophilia A	2017
Tezacaftor and ivacaftor (Symdeko)	CFTR corrector and CFTR potentiator	Cystic fibrosis	2018
Burosumab (Crysvita)	FGF23 antibody	X-linked hypophosphataemia	2018
Cannabidiol (Epidiolex)	Cannabinoid	Dravet syndrome and Lennox–Gastaut syndrome	2018
Binimetinib (Mektovi)	MEK inhibitor	BRAF-mutated melanoma	2018
Encorafenib (Braftovi)	BRAF inhibitor	BRAF-mutated melanoma	2018
Patisiran (Onpattro)	TTR-directed small interfering RNA	Hereditary TTR-mediated amyloidosis	2018
Migalastat (Galafold)	α-galactosidase regulator	Fabry disease	2018
Stiripentol (Diacomit)	GABA reuptake inhibitor	Dravet syndrome	2018
Lanadelumab (Takhzyro)	Kallikrein antibody	Hereditary angioedema	2018
Elapegademase (Revcovi)	Recombinant adenosine deaminase	ADA-SCID	2018
Inotersen (Tegsedi)	TTR-directed antisense oligonucleotide	Hereditary TTR-mediated amyloidosis	2018
Amifampridine (Firdapse)	Potassium channel blocker	Lambert–Eaton myasthenic syndrome	2018
Tafamidis (Vyndaqel)	Transthyretin stabilizer	Transthyretin-mediated amyloidosis cardiomyopathy	2019
Pitolisant (Wakix)	H_3_ receptor antagonist/inverse agonist	Narcolepsy	2019
Afamelanotide (Scenesse)	Melanocortin 1 receptor agonist	Erythropoietic protoporphyria	2019
Tezacaftor, elexacaftor, ivacaftor (Trikafta)	Two CFTR correctors and a CFTR potentiator	Cystic fibrosis	2019
Luspatercept (Reblozyl)	Erythroid maturation agent	Anemia in patients with β- thalassemia	2019
Crizanlizumab (Adakveo)	P-selectin blocker	Sickle cell disease	2019
Givosiran (Givlaari)	ALAS1-directed siRNA	Acute hepatic porphyria	2019
Voxelotor (Oxbryta)	Hemoglobin S polymerization inhibitor	Sickle cell disease	2019
Golodirsen (Vyondys 53)	Exon 53 skipping antisense	Duchenne muscular dystrophy	2019
Selumetinib (Koselugo)	MEK1/2 kinase inhibitor	Neurofibromatosis type 1	2020
Risdiplam (Evrysdi)	SMN2 splicing modifier	Spinal muscular atrophy	2020
Viltolarsen (Viltepso)	Dystrophin splicing modifier	Duchenne muscular dystrophy	2020
Lumasiran (Oxlumo)	HAO1- directed siRNA	Hyperoxaluria type 1	2020
Setmelanotide (Imcivree)	MC_4_ receptor agonist	Rare genetic diseases of obesity	2020
Berotralstat (Orladeyo)	Plasma kallikrein inhibitor	Hereditary angioedema	2020
Inclisiran (Leqvio)	PCSK9-targeted siRNA	To treat heterozygous familial hypercholesterolemia or clinical atherosclerotic cardiovascular disease as an add-on therapy	2021
Vosoritide (Voxzogo)	CNP analogue	To improve growth in children five years of age and older with achondroplasia and open epiphyses	2021
Maralixibat (Livmarli)	IBAT inhibitor	Cholestatic pruritus associated with Alagille syndrome	2021
Belzutifan (Welireg)	HIF-2α inhibitor	Von Hippel-Lindau disease under certain conditions	2021
Avalglucosidase alfa (Nexviazyme)	Recombinant α-glucosidase	Late-onset Pompe disease	2021
Fosdenopterin (Nulibry)	cPMP	Reduce the risk of mortality in molybdenum cofactor deficiency Type A	2021
Casimersen (Amondys 45)	Exon 45-skipping ASO	Duchenne muscular dystrophy	2021
Evinacumab (Evkeeza)	ANGPTL3-targeted mAb	Homozygous familial hypercholesterolemia	2021
Olipudase alfa (Xenpozyme)	Acid sphingomyelinase ERT	Acid Sphingomyelinase Deficiency	2022
Vutrisiran (Amvuttra)	TTR-targeted siRNA	Polyneuropathy of hereditary transthyretin-mediated amyloidosis	2022
Mavacamten (Camzyos)	Cardiac myosin inhibitor	For certain classes of obstructive hypertrophic cardiomyopathy	2022
Ganaxolone (Ztalmy)	GABAA receptor positive allosteric modulator	Seizures in cyclin-dependent kinase-like 5 deficiency disorder	2022
Mitapivat (Pyrukynd)	Pyruvate kinase activator	Hemolytic anemia in pyruvate kinase deficiency	2022
Velmanase alfa (Lamzede)	Recombinant α-mannosidase	Non-central nervous system manifestations of alpha-mannosidosis	2023
Omaveloxolone (Skyclarys)	Mechanism unknown, NRF2 activator	Friedrich’s ataxia	2023
Trofinetide (Daybue)	Mechanism unknown	Rett syndrome	2023
Leniolisib (Joenja)	PI3Kδ inhibitor	Activated PI3Kδ syndrome	2023
Tofersen (Qalsody)	SOD1-targeted ASO	For adults with *SOD1* amyotrophic lateral sclerosis	2023
Pegunigalsidase alfa (Elfabrio)	PEGylated recombinant α-galactosidase Α	Fabry disease	2023
Palovarotene (Sohonos)	Retinoic acid receptor agonist	Reduce the volume of new heterotopic ossification in adults and pediatric patients (aged 8 years and older for females and 10 years and older for males) with fibrodysplasia ossificans progressiva	2023
Pozelimab (Veopoz)	C5-targeted mAb	For patients 1 year old and older with CD55-deficient protein-losing enteropathy (PLE), also known as CHAPLE disease	2023
Cipaglucosidase alfa (Pombiliti)	Recombinant α-glucosidase	Late-onset Pompe disease	2023
Nedosiran (Rivfloza)	LDHA-targeted siRNA	Lower urinary oxalate levels in patients 9 years and older with primary hyperoxaluria type 1 and relatively preserved kidney function	2023
Vamorolone (Agamree)	Corticosteroid	Duchenne muscular dystrophy	2023
Birch triterpenes (Filsuvez)	Mechanism unknown	Wounds associated with dystrophic and junctional epidermolysis bullosa	2023
Eplontersen (Wainua)	TTR-targeted ASO	Polyneuropathy of hereditary transthyretin-mediated amyloidosis	2023
Concizumab (Alhemo)	TFPI-targeted mAb	For routine prophylaxis to prevent bleeding episodes in hemophilia A and B	2024
Deutivacaftor, vanzacaftor and tezacaftor (Alyftrek)	A CFTR potentiator and two CFTR correctors	Cystic fibrosis	2024
Olezarsen (Tryngolza)	APOC-III-directed ASO	Familial chylomicronemia syndrome	2024
Crinecerfont (Crenessity)	CRF type 1 receptor antagonist	Classic congenital adrenal hyperplasia	2024
Marstacimab (Hympavzi)	TFPI-targeted mAb	To prevent or reduce bleeding episodes related to hemophilia A or B	2024
Levacetylleucine (Aqneursa)	Mechanism unknown	Niemann–Pick disease type C	2024
Arimoclomol (Miplyffa)	Mechanism unknown	Niemann–Pick disease type C	2024
Mavorixafor (Xolremdi)	CXC chemokine receptor 4 antagonist	WHIM syndrome (warts, hypogammaglobulinemia, infections and myelokathexis)	2024
Givinostat (Duvyzat)	Histone deacetylase inhibitor	Duchenne muscular dystrophy in individuals aged 6 years and older	2024

Rett syndrome (RTT) is a severe, progressive rare genetic NDD that predominantly affects females, and is one of the leading genetic causes of ID in females.^79^ RTT presents with a distinct natural history characterized by a developmental regression period after seemingly typical early development, followed by the onset of autistic-like features such as intense hand stereotypes, impairment in communication skills, and loss of fine motor skills.^80^ Diagnosis of Rett syndrome is primarily clinical with additional features ranging from epilepsy to growth restrictions, and treatments have long been limited to symptom management. Most individuals with RTT carry loss of function mutations in the X-linked gene methyl-CpG binding protein 2 (*MECP2*), found to be the primary cause of RTT.^81^ In March 2023, the United States Food and Drug Administration (USFDA) authorized Trofinetide--an oral, small molecule, synthetic analog of glycine-proline-glutamate (GPE) able to penetrate the blood-brain barrier--as the first treatment of Rett syndrome for adults and pediatric patients 2 years of age or older.^82^ For patients below a certain age threshold, Trofinetide may reduce the progression of RTT; for those above the age threshold, this treatment may allow neuronal networks to improve in functioning.^80^

Glucose transporter type 1 (GLUT1) deficiency results in deficient glucose transport over the blood-brain barrier due to mutations in the *SLC2A1* gene, leading to reduced glucose availability in the brain and thus causing movement disorders, cognitive impairment, DD, and early onset epilepsy.^83^ In the absence of glucose in the brain, ketones serve as the only alternative energy source. As such, a ketogenic diet (high-fat, low-carbohydrates) is the treatment of choice for GLUT1 deficiency as a mechanism of providing ketones to the brain and restoring brain energy metabolism.^84^ Early initiation of a ketogenic diet would allow for increased cerebral metabolism of the developing brain^11^ and reduction of neuroinflammation in patients, contributing to improved quality of life and decreased risk of comorbidities.^85^ Of note, the long term use of the ketogenic diet can be associated with cardiovascular risks and metabolic disturbances, including changes in BMI, lipid profiles, and metabolic acidosis.^86^

Timothy syndrome (TS) is an extremely rare genetic syndrome primarily characterized by prolonged QT intervals and hand/foot syndactyly alongside multiple system malfunctions, affecting individuals early in life and resulting in severe cardiac arrhythmias.^87^ Caused by heterozygous, gain of function variants in the *CACNA1C* gene, treatments include avoiding QT prolonging medications as well as introducing medications that help maintain normal QT intervals such as beta blockers.^13^ Early diagnosis is of paramount importance in this high-risk patient population.

Smith-Magenis syndrome (SMS) is characterized by distinct facial features that evolve with age, DD and ID, and a typical behavioral phenotype such as maladaptive and self-injurious behaviors alongside significant sleep disturbances.^88^ SMS results from haploinsufficiency of the retinoic acid induced 1 (*RAI1*) gene which maps on the short arm of chromosome 17. Chromosome 17p11.2 microdeletions have been identified as causative in 90% of patients diagnosed with SMS, with the remaining 10% evidenced to have heterozygous pathogenic variants of *RAI1*.^89^ Inversions of typical melatonin secretion have been well-evidenced in patients with SMS in addition to disrupted nocturnal sleep, lending to the considered mechanisms for sleep disturbances in this population. Typically, management of these disturbances requires joint behavioral and medication-based intervention. Tasimelteon, a selective melatonin receptor 1 (MT1) and melatonin receptor 2 (MT2) agonist, was approved by the FDA in 2014 for treatment of individuals with non-24 h sleep/wake disorders and was shown in a double-blind randomized two-period crossover trial of 26 SMS patients to improve total sleep time and quality of sleep.^90^

In these select genetic NDDs, advancements in understanding disease pathogenesis have led to the development of considerable therapeutics to treat co-occurring medical concerns. Furthermore, this insight can inform symptom management across different genetic syndromes where individuals may exhibit similar co-occurring symptoms despite varying genetic diagnoses and etiologies.

Alongside small molecule drugs, advancements in gene therapies have seen an exponential rise over the last decade, encompassing techniques with the potential to alter the expression of genes and therapeutically restore homeostasis.^91^ Angelman syndrome (AS) is a rare genetic NDD characterized by impaired communication skills, ID, and a high prevalence of seizures. AS is caused by insufficient expression of the maternal copy *UBE3A* gene, which is primarily expressed in the brain while the paternal copy is silenced. Several molecular mechanisms can cause AS, such as deletion of the maternal 15q11.2-13.1 region and mutations of the maternal *UBE3A* gene.^92^ Because Angelman syndrome is genetically defined by the loss of functional *UBE3A*, gene therapies that introduce a functional copy of the gene are highly promising. For example, activation of the normally silenced paternal copy of the gene could rescue the absence of functional *UBE3A* in the brain. This approach has been explored through adeno-associated virus (AAV) mediated activation of the paternal UBE3A gene in mouse models, resulting in localized reinstatement of Ube3a to typical levels.^78^ Pharmaceutical companies are currently in preclinical phases exploring therapeutics using such viral vector gene replacement mechanisms.^92^

DNA- or RNA-based targeted therapies such as antisense oligonucleotides (ASOs) have also gained more attention for therapeutic approaches due to their high target sequence recognition specificity.^93^ ASOs work by targeting RNA or DNA to modulate translation, alter splicing, or impact mRNA degradation. For the treatment of epilepsy phenotypes in Angelman syndrome, targeting of the *UBE3A* NAT mRNA antisense transcript that normally silences paternal allele expression for degradation at the transcriptional level is possible, and studies have shown evidence of decreased *UBE3A* NAT expression and rescue of sense *UBE3A* expression in mouse models, in addition to improved cognitive and behavioral phenotypes.^94^ Currently, multiple experimental drugs utilizing NAT-targeting ASOs are in phase 1 or phase 2 clinical trials in adult and pediatric AS patients.^91^

## Challenges to translational approaches for rare neurodevelopmental genetic disorders

Rare genetic disorders, defined as less than 1 in 200,000 in the United States, present unique and extensive challenges for the development of both drug and biologic-based therapeutic interventions. Designing and implementing standard randomized controlled trials are often impossible due to small and typically geographically dispersed patient populations, creating issues in the recruitment of a sufficient number of subjects.^95^ As such, only an estimated 5% of 10,000 recognized rare disease have an approved pharmacotherapy despite millions of affected individuals worlwide.^96^ Clinical practice guidelines for rare disorders have similarly met their own obstacles as a result of limited resources, a lack of published evidence, and small or virtually absent expert groups.^97^ Implementation of the Orphan Drug Act of 1983^98^ facilitated development of therapeutics for rare diseases by incentivizing pharmaceutical companies to pursue therapies for these conditions.^99,100^ Academia partnerships with the pharmaceutical industry have been instrumental in facilitating development of most of these orphan drugs.^101^ Although the current model of drug development for rare diseases has led to the approval of many drugs through various therapeutic modalities, this model is not always feasible for very small populations, especially in cases where target populations are as small as one individual.^102^ For such instances, an “*N*-of-1” approach has shown increasing potential as a model for developing individualized, genetically targeted therapies used by only one or very few people with a specific rare condition. These *N*-of-1 genetic therapies are usually designed to restore a protein’s function or reduce the expression of a dysfunctional protein, targeting the root cause of the disease.

Individualized outcome assessments and biomarkers that can reliably and objectively measure clinical impacts relevant to the patient and their underlying condition should similarly be considered when designing *N*-of-1 trials.^103^ However, drug trials of the CNS have met repeated failures due to insufficient modulation of the intended target, highlighting the need for additional research focused on identifying CNS biomarkers.^104^ Establishing dose-dependent CNS effects requires one or more quantitative CNS measures of a drug’s effect on brain function to allow effective interpretation of clinical results informing mechanisms and future dose selection in efficacy trials. Due in part to the difficulty of identifying precise, biologically relevant markers, many drugs that have met preclinical success fail to translate to clinical outcomes. Furthermore, regulatory hurdles and ethical considerations have limited the number of successful clinical trials^103,105^ combined with the small patient populations for rare diseases which requires novel flexible clinical trial design approaches, for example, adaptive trials that allow modifications on the study design based on accumulating data.^106^

### Failed clinical trials for neurobehavioral endpoints

Fragile X syndrome (FXS) is a NDD caused by the full silencing of the Fragile X Messenger Ribonucleoprotein 1 (*FMR1*) gene and is the most commonly known genetic cause of ASD and inherited intellectual disabilities.^107^ Silencing of *FMR1*, which normally encodes the Fragile X Messenger Ribonucleoprotein (FMRP), leads to subsequent loss FMRP function which regulates the synthesis of multiple proteins involved in synaptic functioning.^104^ *Fmr1*-knockout mice have been used in preclinical studies to evaluate the efficacy of two primary targets: group 1 metabotropic glutamate receptors (mGluRs) and gamma amino-butyric acid (GABA) receptors.^108^ Despite preclinical results demonstrating rescue of functional, morphological, and behavioral deficits in rodents,^104^ several clinical trials of two mGluR5-targeting therapies in adults with FXS failed to show statistically significant differences from placebo utilizing efficacy outcomes based on primary caregiver-rated measures.^109^

Questions have emerged about whether these clinical trials utilized the optimal outcome measures. Notably, phenotypes addressed in *Fmr1-*knockout mice such as rate of protein synthesis, audiogenic seizures, and spine morphology have not been explored clinically due to inaccessible or unattainable readouts, and other utilized preclinical readouts such as self-grooming are not reliable proxies of human symptoms.^108^ Additional measures that can be broadly applied to both preclinical models and clinically as part of therapy trials, such as EEG signatures, may be needed to improve translation.^110^

The RASopathies are a group of disorders characterized by germline mutations in one or more genes encoding a component of the RAS/MAPK pathway, resulting in increased signaling.^111^ Among the RASopathies, neurofibromatosis type 1 (NF1) and Noonan syndrome (NS) have been explored in both preclinical and clinical trials. Statins, or 3-hydroxy-3-methylglutaryl coenzyme A (HMG-CoA) reductase inhibitors, inhibit the synthesis of substrates needed for prenylation which is required for activation of RAS at the cellular level, serving as the focus of many NF1 and NS trials.^112^ The efficacy of statins has been reported in both NF1 mice, for improved synaptic plasticity and cognitive function, as well as NS mice for the treatment of memory and learning deficits. Translating these preclinical findings to the clinic required several randomized placebo-controlled trials exploring the efficacy of statins for improving cognitive function that were conducted but saw minimal success.^113^ Both lovastatin, the first commercially available statin, and simvastatin, a second-generation statin, showed potential benefits in smaller trials for NF1, but failed to show efficacy in primary outcome measures in three large randomized controlled trials evaluating cognition and behavior in children with NF1.^112^ Clinical results for recently completed NS trials utilizing statins have yet to be released at the time of writing.

PTEN hamartoma tumor syndrome (PHTS) characterizes a group of inherited syndromes caused by loss of function germline *PTEN* mutations, resulting in its loss of function as a tumor suppressor.^114^ Loss of PTEN function upregulates the PI3K/AKT/MTOR signaling pathway, providing a potential avenue for pharmacological inhibition at different points of the pathway. Neurobehavioral symptoms in PHTS have been suggested to respond to treatment through mTOR inhibitors as reported in preclinical studies investigating the rescue of neuronal hypertrophy and autistic-like behavioral abnormalities in mice with *Pten* deletions.^115^ However, clinical studies examining the efficacy and safety of mTOR inhibitors on behavior and cognition have been limited. A phase-2, double-blinded, placebo-controlled study investigating the mTOR inhibitor everolimus on a variety of behavioral and cognitive endpoints in humans with PHTS was conducted but failed to demonstrate intervention-related improvements in primary outcome measures.^115^ Promise for future opportunities were highlighted as secondary efficacy endpoints moved towards the direction of improvement.

### Lessons learned

As previously mentioned, a common factor inhibiting the success of therapeutics in clinical trials for NDDs results from the difficulty for drugs to permeate the blood-brain barrier. While protecting the brain from most blood-borne substances, the BBB excludes approximately 98% of small molecule drugs and all macromolecular therapeutics from accessing the brain.^69^ When considering efficacy criterion for therapies in patient cohorts, the ability to penetrate the BBB and generate dose-dependent effects on the CNS is necessary and, likewise, challenging to discern with certainty in preclinical studies utilizing animal models.

Recent findings on the mechanistic similarities between genes associated in autism and cancer predisposition may provide insights addressing disparate challenges with similar therapeutic approaches.^116^ Elevated frequencies of heterozygous pathogenic variants in genes associated with both NDDs and somatic cancers, including *PTEN*, provide a framework to suggest that these recurrent mutations serve as a key component in the shared ASD and cancer etiology.^117^

Findings from clinical trials and cancer have highlighted the need for combination therapies to address and overcome the emergence of single drug resistance. For cancer to respond, different and combined medications are needed to target multiple gene changes underlying that cancer. The same approach has been attributed to the various gene variants underlying rare genetic NDDs and their corresponding mechanisms in the brain.^36,118^ A therapeutic approach incorporating combinations of medications targeting the same signaling pathway or combining medications with behavioral interventions may be necessary in cases where a therapeutic intervention leads to compensatory feedback loops, thus insuring targeting of a signal along the entirety of its pathway.^119^ Many medications have similarly shown promise in their early stages of intake but lose efficacy over time. In such cases, combination medications may serve as a solution.^120^

## Impact and ethical considerations of rare disease therapeutics on common neurodevelopmental phenotypes

Individuals with NDDs face several challenges to their physical health. Co-occurring medical conditions like seizures, sleep disorders, gastrointestinal, immunological, musculoskeletal, and psychiatric disproportionately impact neurodiverse individuals.^121,122^ Given the high prevalence of NDDs, most individuals with neurodevelopmental phenotypes likely represent varying degrees of polygenic risk. This genomic complexity makes small molecules and gene therapies developed for rare genetic syndromes of limited clinical utility within larger groups. This genetic heterogeneity also marks a major challenge in developing targeted interventions for co-occurring medical concerns. Therefore, unique approaches will be required to translate the vast efforts in place developed for rare diseases to impact those with polygenic risk genomic profiles. This is particularly relevant as the promise of polygenic risk scores in predicting disease risk and opportunities for therapy appears to be complex, specifically for common medical conditions like cardiovascular disease.^123^ The development of therapies with applicability to multiple genetic syndromes or groups of disorders based on shared epistatic interactions or similar mechanistic impacts on cellular/systems functions may serve as a window for broader therapeutic applicability. A few examples are emerging of these approaches. For example, therapeutics approved for the treatment of Rett syndrome may have broader applicability to other genetic syndromes based on its effect on neuronal synaptic and inflammation.^81^ Similarly, small molecules like MTOR inhibitors may have broader applicability across many genetic etiologies that all intersect in downstream hyperactivation of this pathway and due to its broad effects in modulating synaptic growth, plasticity and behavior.

Ethical challenges are also emerging surrounding the use of gene therapies in NDDs. The potential use of these therapies for cognitive enhancement and prenatal eugenics, coupled with off target effects, genetic mosaicism, germline transmission risks, and challenges with informed consent and barriers to access due to costs remain unaddressed challenges as the demand for therapeutic interventions increases with the expansion of novel genetic conditions. Coupled with the technical uncertainties of the specific gene therapy approach utilized and the need for long-term surveillance of clinical outcomes, the financial burden to the clinical and research infrastructure remain unsolved challenges.^124–127^ The challenge of broad therapeutic applicability in the larger population of individuals affected by NDDs also raises ethical questions about target of these therapies. Harnessing the innate plasticity of the nervous system to promote changes in behavioral circuits and networks through therapeutics could open opportunities for misuse. As the field of neurogenetics accelerates the pace of translating basic discoveries into therapeutic interventions, community conversation has emerged surrounding their potential impact on altering intellectual capacity and behavioral diversity. For many, the challenges these therapies face with access to the CNS facilitates refocusing their efficacy to addressing their impacts on treating co-occurring diagnoses associated with significant morbidity and quality of life rather than on a cure for their cognitive and behavioral phenotypes. These conversations occur in parallel with initiatives that seek a rigorous ethics-informed approach to the participation of research studies that use genomics information.^128^ Integrating community involvement as these advances translate to the clinic will be essential to ensure that the needs of those most impacted by health disparities associated with neurodevelopmental disabilities are met.

## Data Availability

Data sharing is not applicable to this article as no datasets were generated or analyzed during the current study. All data generated or analyzed during this study are included in this published article and its supplementary information files.
